# Dimethyl 2,6-dihydroxy­benzene-1,4-dicarboxyl­ate

**DOI:** 10.1107/S1600536810011074

**Published:** 2010-03-31

**Authors:** Deming Zhao, Kun Wang, Jianting Zhang, Ningren Jin

**Affiliations:** a College of Chemical Engineering and Materials Science, Zhejiang University of Technology, Hangzhou 310014, People’s Republic of China

## Abstract

The title compound, C_10_H_10_O_6_, was obtained from an esterification reaction of 2,6-dihydroxy­terephthalic acid and methanol. In the mol­ecular structure, all of the C atoms are nearly coplanar. The two hydr­oxy groups have *C*2 symmetry. Intra­molecular O—H⋯O hydrogen bonds are observed. In the crystal, weak O—H⋯O inter­actions link the mol­ecules.

## Related literature

For general background to terephthalate derivatives, see: Brunner (1928 ▶); Teruhiko *et al.* (1998 ▶). For a related structure, see: Dai *et al.* (2005 ▶).
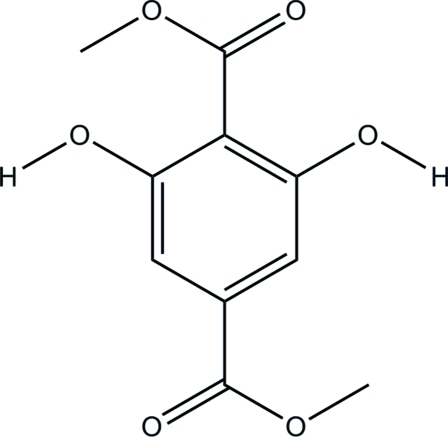


## Experimental

### 

#### Crystal data

C_10_H_10_O_6_
*M* *_r_* = 226.18
Monoclinic, 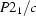

*a* = 11.6462 (8) Å
*b* = 7.0925 (3) Å
*c* = 13.5745 (10) Å
β = 114.327 (9)°
*V* = 1021.70 (11) Å^3^
*Z* = 4
Mo *K*α radiation
μ = 0.12 mm^−1^
*T* = 293 K
0.34 × 0.26 × 0.20 mm

#### Data collection

Oxford Diffraction Xcalibur Eos Gemini ultra diffractometer
Absorption correction: multi-scan (*CrysAlis PRO*; Oxford Diffraction, 2009 ▶) *T*_min_ = 0.865, *T*_max_ = 1.000
4572 measured reflections
2083 independent reflections
1161 reflections with *I* > 2σ(*I*)
*R*_int_ = 0.023

#### Refinement

*R*[*F* ^2^ > 2σ(*F* ^2^)] = 0.049
*wR*(*F* ^2^) = 0.178
*S* = 1.09
2083 reflections
149 parameters
H-atom parameters constrained
Δρ_max_ = 0.26 e Å^−3^
Δρ_min_ = −0.21 e Å^−3^

### 

Data collection: *CrysAlis PRO* (Oxford Diffraction, 2009 ▶); cell refinement: *CrysAlis PRO*; data reduction: *CrysAlis PRO*; program(s) used to solve structure: *SHELXS97* (Sheldrick, 2008 ▶); program(s) used to refine structure: *SHELXL97* (Sheldrick, 2008 ▶); molecular graphics: *OLEX2* (Dolomanov *et al.*, 2009 ▶); software used to prepare material for publication: *OLEX2*.

## Supplementary Material

Crystal structure: contains datablocks global, I. DOI: 10.1107/S1600536810011074/gw2078sup1.cif

Structure factors: contains datablocks I. DOI: 10.1107/S1600536810011074/gw2078Isup2.hkl

Additional supplementary materials:  crystallographic information; 3D view; checkCIF report

## Figures and Tables

**Table 1:** Hydrogen-bond geometry (Å, °)

*D*—H⋯*A*	*D*—H	H⋯*A*	*D*⋯*A*	*D*—H⋯*A*
O3—H3⋯O2	0.82	1.84	2.567 (3)	147
O4—H4⋯O1	0.82	1.89	2.593 (3)	144
O4—H4⋯O3^i^	0.82	2.58	3.099 (3)	123

Symmetry code: (i) 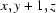.

**Fig. 1.**
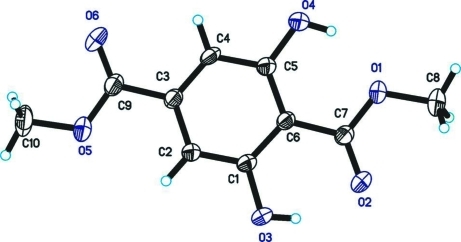
The asymmetric unit of the structure of the title compound, with the atomic labeling scheme. Displacement ellipsoids are drawn at the 50% probability level.

**Fig. 2.**
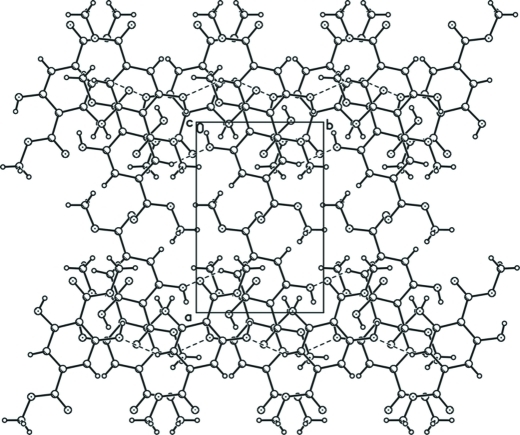
The molecule packing of the title compound showing O—H···O interactions.

## supplementary crystallographic information

### Comment

The title compound as one of terephthalate derivatives, is an important pharmacological and material intermediate (Brunner, 1928; Teruhiko *et al.*, 1998). There is almost no report about crystal structure. As part of our ongoing studies, we now describe the synthesis and the crystal structure of the title compound. In this paper, the crystal structure of it was determined (Fig. 1). The molecule contains benzene ring, dihydroxygroup and dimethyl group. All of atoms except the hydrogen atoms are nearly coplanar. The terminal dimethyl group are centrosymmetric. The dihydroxy group are axisymmetric. In the crystal structure, intramolecular O—H···O hydrogen bonds are observed.

### Experimental

2,6-dihydroxyterephthalic acid (15 mmol) was dissolved in methanol (45 ml), thionyl dichloride (3 ml) was slowly added to the methanol solution afterward, and the mixture was stired at reflux temperature for 72 hours (monitored by TLC). Then the solvent was distilled under vacuum, and the residue was poured into water (50 ml). The pH of the solution was adjusted with sodium bicarbonate to pH = 7.0. The resulting white solid was filtered off, washed with water. The obtained solid was dissolved in methanol. Single crystals were obtained by slow evaporation of a methanol solution.

### Refinement

H atoms were placed in calculated position with C—H=0.96 (1) Å(sp3), C—H=0.93 Å(aromatic). All H atoms included in the final cycles of refinement as riding mode, with U_iso_(H)=1.2U_eq_ of the carrier atoms.

### Crystal data

**Table:**

C_10_H_10_O_6_	*F*(000) = 472
*M**_r_* = 226.18	*D*_x_ = 1.470 Mg m^−^^3^
Monoclinic, *P*2_1_/*c*	Mo *K*α radiation, λ = 0.71073 Å
Hall symbol: -P 2ybc	Cell parameters from 1855 reflections
*a* = 11.6462 (8) Å	θ = 2.9–29.2°
*b* = 7.0925 (3) Å	µ = 0.12 mm^−^^1^
*c* = 13.5745 (10) Å	*T* = 293 K
β = 114.327 (9)°	Block, white
*V* = 1021.70 (11) Å^3^	0.34 × 0.26 × 0.20 mm
*Z* = 4	

### Data collection

**Table:**

Oxford Diffraction Xcalibur Eos Gemini ultra diffractometer	2083 independent reflections
Radiation source: fine-focus sealed tube	1161 reflections with *I* > 2σ(*I*)
graphite	*R*_int_ = 0.023
Detector resolution: 16.0395 pixels mm^-1^	θ_max_ = 26.4°, θ_min_ = 3.3°
ω scans	*h* = −14→10
Absorption correction: multi-scan (*CrysAlis PRO*; Oxford Diffraction, 2009)	*k* = −8→8
*T*_min_ = 0.865, *T*_max_ = 1.000	*l* = −16→15
4572 measured reflections	

### Refinement

**Table:**

Refinement on *F*^2^	Primary atom site location: structure-invariant direct methods
Least-squares matrix: full	Secondary atom site location: difference Fourier map
*R*[*F*^2^ > 2σ(*F*^2^)] = 0.049	Hydrogen site location: inferred from neighbouring sites
*wR*(*F*^2^) = 0.178	H-atom parameters constrained
*S* = 1.09	*w* = 1/[σ^2^(*F*_o_^2^) + (0.0906*P*)^2^ + 0.0929*P*] where *P* = (*F*_o_^2^ + 2*F*_c_^2^)/3
2083 reflections	(Δ/σ)_max_ = 0.002
149 parameters	Δρ_max_ = 0.26 e Å^−^^3^
0 restraints	Δρ_min_ = −0.20 e Å^−^^3^

### Special details

**Table:**

Geometry. All esds (except the esd in the dihedral angle between two l.s. planes) are estimated using the full covariance matrix. The cell esds are taken into account individually in the estimation of esds in distances, angles and torsion angles; correlations between esds in cell parameters are only used when they are defined by crystal symmetry. An approximate (isotropic) treatment of cell esds is used for estimating esds involving l.s. planes.
Refinement. Refinement of *F*^2^ against ALL reflections. The weighted *R*-factor wR and goodness of fit *S* are based on *F*^2^, conventional *R*-factors *R* are based on *F*, with *F* set to zero for negative *F*^2^. The threshold expression of *F*^2^ > σ(*F*^2^) is used only for calculating *R*-factors(gt) etc. and is not relevant to the choice of reflections for refinement. *R*-factors based on *F*^2^ are statistically about twice as large as those based on *F*, and *R*- factors based on ALL data will be even larger.

### Fractional atomic coordinates and isotropic or equivalent isotropic displacement parameters (Å^2^)

**Table:**

	*x*	*y*	*z*	*U*_iso_*/*U*_eq_	
O1	1.10292 (17)	0.3212 (3)	0.65789 (15)	0.0531 (6)	
O2	1.16658 (17)	0.0296 (3)	0.71430 (17)	0.0594 (6)	
O3	1.00830 (17)	−0.2437 (3)	0.65185 (18)	0.0588 (6)	
H3	1.0760	−0.1914	0.6860	0.088*	
O4	0.8686 (2)	0.3940 (3)	0.5366 (2)	0.0694 (7)	
H4	0.9419	0.4218	0.5747	0.104*	
O5	0.48877 (19)	−0.0097 (4)	0.36441 (19)	0.0809 (8)	
O6	0.55844 (19)	−0.2972 (3)	0.42310 (17)	0.0622 (6)	
C1	1.2310 (3)	0.3910 (5)	0.7143 (3)	0.0605 (9)	
H1A	1.2712	0.3957	0.6652	0.091*	
H1C	1.2773	0.3082	0.7733	0.091*	
H1B	1.2290	0.5152	0.7417	0.091*	
C2	1.0826 (2)	0.1371 (4)	0.6627 (2)	0.0418 (6)	
C3	0.9508 (2)	0.0794 (3)	0.6008 (2)	0.0370 (6)	
C4	0.9197 (2)	−0.1122 (3)	0.5979 (2)	0.0393 (6)	
C5	0.7983 (2)	−0.1768 (4)	0.5401 (2)	0.0395 (6)	
H5	0.7798	−0.3044	0.5397	0.047*	
C6	0.7048 (2)	−0.0498 (4)	0.4829 (2)	0.0391 (6)	
C7	0.7314 (2)	0.1397 (4)	0.4830 (2)	0.0449 (7)	
H7	0.6675	0.2237	0.4438	0.054*	
C8	0.8531 (2)	0.2059 (3)	0.5412 (2)	0.0430 (7)	
C9	0.5725 (3)	−0.1122 (4)	0.4170 (2)	0.0476 (7)	
C10	0.4355 (3)	−0.3758 (5)	0.3582 (3)	0.0769 (11)	
H10A	0.4105	−0.3402	0.2840	0.115*	
H10B	0.3752	−0.3286	0.3835	0.115*	
H10C	0.4392	−0.5108	0.3641	0.115*	

### Atomic displacement parameters (Å^2^)

**Table:**

	*U*^11^	*U*^22^	*U*^33^	*U*^12^	*U*^13^	*U*^23^
O1	0.0335 (11)	0.0493 (12)	0.0635 (13)	−0.0079 (9)	0.0067 (10)	−0.0032 (10)
O2	0.0326 (10)	0.0563 (12)	0.0718 (14)	0.0062 (9)	0.0039 (10)	0.0043 (11)
O3	0.0360 (11)	0.0445 (11)	0.0775 (14)	0.0103 (9)	0.0048 (10)	0.0115 (11)
O4	0.0480 (13)	0.0347 (11)	0.0953 (18)	0.0029 (9)	−0.0010 (12)	0.0016 (11)
O5	0.0340 (11)	0.0803 (17)	0.0952 (19)	0.0052 (12)	−0.0068 (12)	0.0146 (14)
O6	0.0367 (11)	0.0582 (14)	0.0749 (15)	−0.0086 (10)	0.0062 (10)	−0.0071 (11)
C1	0.0382 (16)	0.070 (2)	0.0634 (19)	−0.0148 (15)	0.0108 (15)	−0.0065 (17)
C2	0.0343 (14)	0.0434 (16)	0.0428 (15)	0.0023 (12)	0.0111 (12)	−0.0003 (13)
C3	0.0298 (13)	0.0377 (14)	0.0389 (14)	0.0039 (11)	0.0094 (11)	−0.0029 (11)
C4	0.0333 (14)	0.0376 (15)	0.0430 (15)	0.0079 (11)	0.0116 (12)	0.0029 (12)
C5	0.0341 (15)	0.0331 (13)	0.0464 (15)	0.0031 (11)	0.0116 (13)	−0.0024 (12)
C6	0.0312 (14)	0.0421 (15)	0.0390 (14)	0.0021 (11)	0.0096 (12)	−0.0025 (11)
C7	0.0298 (14)	0.0459 (17)	0.0460 (16)	0.0130 (12)	0.0026 (12)	0.0021 (13)
C8	0.0366 (15)	0.0346 (15)	0.0498 (16)	0.0029 (11)	0.0097 (13)	−0.0027 (12)
C9	0.0322 (15)	0.0552 (19)	0.0487 (17)	0.0008 (14)	0.0098 (13)	−0.0040 (14)
C10	0.0425 (19)	0.090 (3)	0.084 (2)	−0.0231 (18)	0.0110 (18)	−0.024 (2)

### Geometric parameters (Å, °)

**Table:**

O1—C2	1.334 (3)	C2—C3	1.472 (4)
O1—C1	1.454 (3)	C3—C4	1.403 (3)
O2—C2	1.210 (3)	C3—C8	1.413 (3)
O3—C4	1.360 (3)	C4—C5	1.382 (3)
O3—H3	0.8200	C5—C6	1.379 (3)
O4—C8	1.351 (3)	C5—H5	0.9300
O4—H4	0.8200	C6—C7	1.379 (4)
O5—C9	1.190 (3)	C6—C9	1.495 (4)
O6—C9	1.329 (3)	C7—C8	1.389 (4)
O6—C10	1.449 (3)	C7—H7	0.9300
C1—H1A	0.9600	C10—H10A	0.9600
C1—H1C	0.9600	C10—H10B	0.9600
C1—H1B	0.9600	C10—H10C	0.9600
			
C2—O1—C1	118.1 (2)	C6—C5—H5	120.4
C4—O3—H3	109.5	C4—C5—H5	120.4
C8—O4—H4	109.5	C7—C6—C5	120.8 (2)
C9—O6—C10	117.3 (3)	C7—C6—C9	117.7 (2)
O1—C1—H1A	109.5	C5—C6—C9	121.6 (2)
O1—C1—H1C	109.5	C6—C7—C8	120.4 (2)
H1A—C1—H1C	109.5	C6—C7—H7	119.8
O1—C1—H1B	109.5	C8—C7—H7	119.8
H1A—C1—H1B	109.5	O4—C8—C7	115.6 (2)
H1C—C1—H1B	109.5	O4—C8—C3	124.2 (2)
O2—C2—O1	121.8 (2)	C7—C8—C3	120.3 (2)
O2—C2—C3	124.1 (2)	O5—C9—O6	123.3 (3)
O1—C2—C3	114.1 (2)	O5—C9—C6	124.5 (3)
C4—C3—C8	117.4 (2)	O6—C9—C6	112.2 (2)
C4—C3—C2	118.8 (2)	O6—C10—H10A	109.5
C8—C3—C2	123.8 (2)	O6—C10—H10B	109.5
O3—C4—C5	116.8 (2)	H10A—C10—H10B	109.5
O3—C4—C3	121.3 (2)	O6—C10—H10C	109.5
C5—C4—C3	121.9 (2)	H10A—C10—H10C	109.5
C6—C5—C4	119.3 (2)	H10B—C10—H10C	109.5

### Hydrogen-bond geometry (Å, °)

**Table:**

*D*—H···*A*	*D*—H	H···*A*	*D*···*A*	*D*—H···*A*
O3—H3···O2	0.82	1.84	2.567 (3)	147
O4—H4···O1	0.82	1.89	2.593 (3)	144
O4—H4···O3^i^	0.82	2.58	3.099 (3)	123

Symmetry codes: (i) *x*, *y*+1, *z*.
